# P94 The microbiota of the hospital environment

**DOI:** 10.1093/jacamr/dlaf230.101

**Published:** 2025-12-04

**Authors:** Danielle Weaver, Alexandra Verhey, Oluwafunmilayo Adungba, Luke Ward, Michelle Worsley, Rajesh Rajendran, Christopher Smith, Tim Felton

**Affiliations:** Research and Innovation, Manchester University NHS Foundation Trust, Manchester, UK; Division of Infection Immunity and Respiratory Medicine, University of Manchester, Manchester, UK; Research and Innovation, Manchester University NHS Foundation Trust, Manchester, UK; Research and Innovation, Manchester University NHS Foundation Trust, Manchester, UK; Research and Innovation, Manchester University NHS Foundation Trust, Manchester, UK; Infection prevention and control, Manchester University NHS Foundation Trust, Manchester, UK; Infection prevention and control, Manchester University NHS Foundation Trust, Manchester, UK; Research and Innovation, Manchester University NHS Foundation Trust, Manchester, UK; Research and Innovation, Manchester University NHS Foundation Trust, Manchester, UK; Division of Infection Immunity and Respiratory Medicine, University of Manchester, Manchester, UK

## Abstract

**Background:**

Healthcare-associated infections (HAIs) are a significant public health issue. HAIs cause extended hospital stays, increased mortality, reduced quality of life and cost the NHS an estimated £2.7 Billion annually. In 2023, the most reported HAI bacterial pathogens in England were *Escherichia coli* (16.5%) and *Staphylococcus aureus* (10.6%). In healthcare, infection prevention and control (IPC) teams use evidence-based interventions to control the spread of infectious agents. The built environment is a potential reservoir for pathogens, and surfaces frequently touched by healthcare workers, patients and/or visitors are known to play a role in HAI transmission. To minimize infection spread, IPC cleaning protocols routinely include hydrogen peroxide fumigation (known as Deprox) to decontaminate a room following discharge of a patient with an infection. Commercial adenosine triphosphate (ATP) luminometers are widely used to monitor cleaning protocols in clinical settings. However, ATP detection methods have limitations including no clear industry standards and the ability to detect a variety of biological contaminants (e.g. non-pathogenic microorganisms and organic soil).

**Objectives and methods:**

To explore the hospital environmental microbiome, we performed ATP detection, 16S rRNA sequencing and quantitative PCR on various sampling sites (door, floor, sink, bed tray and wall) in side-rooms shortly after patient stays. In addition, for a subset of rooms we analysed two sample areas before and after Deprox, to assess the impact of decontamination on the environmental microbiome.

**Results:**

Bacterial burden data was used to inform quality control for the microbiome analyses, and 64% of samples were defined as having a significant microbiome. A few taxa were found consistently in all areas sampled: *Escherichia-Shigella, Staphylococcus, Corynebacterium* and *Streptococcus. Acinetobacter* was identified in all sample areas, excluding wall samples. Some taxa appeared to be more predominant in one sampling site, including *Bacteroides* in floor samples, and *Methylobacterium* in sink samples. ATP levels and microbiome diversity varied significantly between the areas sampled, with the highest levels found in floor then bed-tray samples. Bacterial burden was also highest in floor samples. ATP levels dropped significantly following the decontamination procedure and 70% of samples were below a ‘strict’ RLU cutoff of 250 (all were below a commonly used RLU cutoff of 500). Although not significant, there were reductions in bacterial burden and microbiome diversity following Deprox. However, 37% of samples had a significant microbiome remaining after Deprox. Overall, ATP levels did not significantly correlate with bacterial burden or microbiome diversity.

**Conclusions:**

Although it gives a useful indication of surface cleanliness, ATP monitoring does not give a clear picture of the bacteria present in the environment. Molecular analyses provided a detailed insight into the bacterial burden and specific Genera present in the environment and identified pathogen-containing Genera in all sample sites tested. As ATP levels did not correlate with bacterial burden or diversity, the viability of the potential pathogenic bacteria in the environment is unclear. Future studies are needed to investigate the viability of common pathogens in the hospital environment and further our understanding of the potential environmental reservoir for HAIs.

